# Effect of beta blocker use and type on hypoglycemia risk among hospitalized insulin requiring patients

**DOI:** 10.1186/s12933-019-0967-1

**Published:** 2019-11-27

**Authors:** Kathleen Dungan, Jennifer Merrill, Clarine Long, Philip Binkley

**Affiliations:** 1 Division of Endocrinology, Diabetes & Metabolism, The Ohio State University, 5th Floor McCampbell Hall, 1581 Dodd Drive, Columbus, OH 43210-1296 USA; 2 Division of Endocrinology, Duke University, 30 Duke Medicine Circle, Durham, NC 22710 USA; 3 The Ohio State University College of Medicine, 370 W. 9th Ave, Columbus, OH 43210 USA; 4 Division of Cardiovascular Medicine, The Ohio State University, 452 W. 10th Ave, Columbus, OH 43210 USA

**Keywords:** Hypoglycemia, Beta blocker, Diabetes mellitus, Hospitalized patients, Mortality

## Abstract

**Background:**

Although beta blockers could increase the risk of hypoglycemia, the difference between subtypes on hypoglycemia and mortality have not been studied. This study sought to determine the relationship between type of beta blocker and incidence of hypoglycemia and mortality in hospitalized patients.

**Methods:**

We retrospectively identified non-critically ill hospitalized insulin requiring patients who were undergoing bedside glucose monitoring and received either carvedilol or a selective beta blocker (metoprolol or atenolol). Patients receiving other beta blockers were excluded. Hypoglycemia was defined as any glucose < 3.9 mmol/L within 24 h of admission (Hypo_1day_) or throughout hospitalization (Hypo_T_) and any glucose < 2.2 mmol/L throughout hospitalization (Hypo_severe_).

**Results:**

There were 1020 patients on carvedilol, 886 on selective beta blockers, and 10,216 on no beta blocker at admission. After controlling for other variables, the odds of Hypo_1day,_ Hypo_T_ and Hypo_severe_ were higher for carvedilol and selective beta blocker recipients than non-recipients, but only in basal insulin nonusers. The odds of Hypo_1day_ (odds ratio [OR] 1.99, 95% confidence interval [CI] 1.28, 3.09, p = 0.0002) and Hypo_T_ (OR 1.38, 95% CI 1.02, 1.86, p = 0.03) but not Hypo_severe_ (OR 1.90, 95% CI 0.90, 4.02, p = 0.09) were greater for selective beta blocker vs. carvedilol recipients in basal insulin nonusers. Hypo_1day_, Hypo_T_, and Hypo_severe_ were all associated with increased mortality in adjusted models among non-beta blocker and selective beta blocker recipients, but not among carvedilol recipients.

**Conclusions:**

Beta blocker use is associated with increased odds of hypoglycemia among hospitalized patients not requiring basal insulin, and odds are greater for selective beta blockers than for carvedilol. The odds of hypoglycemia-associated mortality are increased with selective beta blocker use or nonusers but not in carvedilol users, warranting further study.

## Background

The prevalence of hypoglycemia (defined as blood glucose < 3.9 mmol/L or 70 mg/dL) is estimated to be 4.2% of all blood glucose measurements, or 3.5% of patient days in non-critically ill hospitalized patients in the United States [1]. Hypoglycemia is even more common among insulin-treated hospitalized patients [2]. Age, insulin dose, mistimed insulin doses, low body mass index, impaired renal function, and changes in diet or concomitant medications such as steroids are all potential contributing factors to hypoglycemic episodes among hospitalized patients with diabetes [3–5]. While the precise impact of iatrogenic hypoglycemia on hospital outcomes is debated, hypoglycemia is associated with increased length of stay, mortality and cost [6, 7].

Beta blockers (BB) are an essential component of guideline directed therapy for patients with heart failure and coronary artery disease, and are frequently used to treat hypertension. BB therapy is purported to increase the risk of severe or prolonged hypoglycemia by blunting the early adrenergic symptoms of impending hypoglycemia [8, 9]. In particular, nonselective BB cause concern due to blockade of catecholamine-induced arterial vasodilation mediated by β2 receptors resulting in unopposed α-receptor stimulation during hypoglycemia [10, 11]. However, other literature supports the general safety of β-1 selective blockade (SBB) in patients with diabetes [12]. In fact, BBs including the nonselective agent carvedilol, have been shown to prevent the expected impaired autonomic response to hypoglycemia known to follow an antecedent hypoglycemic event [13, 14]. Moreover, in the presence of severe hypoglycemia, BB may decrease the risk of hypoglycemia-associated arrhythmias and death [15, 16].

However, there are few studies assessing the risk of BB use in acutely ill patients, in whom awareness of and counterregulatory responses to hypoglycemia may be impaired [17]. BBs have been associated with increased risk for cardiovascular events in patients with diabetes and heart disease, but it is unclear whether hypoglycemia or glucose lowering therapy plays a role [18]. In addition, many studies analyze the frequency of severe hypoglycemia, while non-severe events or even hypoglycemia unawareness can also have significant impact on well-being. Finally, it is unknown whether hypoglycemia risk and consequences differ by commonly used beta–blocker types. A large clinical trial that compared the metabolic effects of metoprolol (a selective inhibitor of β1 receptors) to carvedilol (which inhibits β1, β2, and α1 receptors), carvedilol demonstrated more favorable effects on glycemic control and fewer hypoglycemia symptoms, though the authors note that the latter may be better explained by factors other than hypoglycemia [19]. Thus, the objective of this study is to determine the relationship between BB use and type with hypoglycemia and hypoglycemia-mediated mortality among hospitalized patients.

## Methods

### Patient selection

Patients over 18 years of age who were hospitalized between January 1, 2014 and December 31, 2015 with at least two blood glucoses per day (point of care or serum) and an order for subcutaneous insulin were identified from the study institution’s Information Warehouse. The Information Warehouse is a data analysis tool that validates patient information from multiple sources. Exclusion criteria included intravenous insulin infusion, admission directly to the intensive care unit, or pregnancy. Intensive care settings were excluded as insulin is most often delivered intravenously in accordance with American Diabetes Association (ADA) standards of care [20]. Pregnant patients were excluded because of lower glycemic targets which are not generalizable to the rest of the inpatient population [21]. In order to accurately attribute risk and to distinguish risk between BB types, patients who initiated BB therapy after admission, switched BB type during admission, or received a nonselective BB other than carvedilol were excluded. For patients with repeat admissions, one admission was chosen randomly for inclusion into the analysis. This was done to decrease potential bias and better capture the general inpatient population. Patients who have been admitted multiple times during a 2 year period are likely to be significantly more debilitated than a patient being admitted for the first time. The Ohio State University Institutional Review Board approved all study procedures.

### Clinical and laboratory variables

Demographics, body mass index, Hemoglobin A1c during or within 30 days prior to admission, BB at admission, initial serum creatinine, service type (medical, surgical, cardiac), and concomitant medications were collected. Due to anticipated differential use of carvedilol vs. other BB, heart failure was identified via the International Classification of Diseases (ICD), ICD-9 of 428.xx or ICD-10 of I50.xx). Diabetes diagnosis was identified as an ICD-9 250.xx or ICD-10 E08-13.xxxx on the active problem list. Basal insulin administration was defined as any order for glargine, detemir, NPH, 70/30, 75/25, or degludec at admission. In-hospital mortality was also collected as a clinical outcome.

Point of care and serum glucoses were collected and mean glucose was reported at admission, 24 and 72 h. Definitions of hypoglycemia included glucose < 3.9 mmol/L (70 mg/dL) within 24 h of admission (Hypo_1day_), or throughout the hospitalization (Hypo_T_). Severe hypoglycemia was defined as glucose < 2.2 mmol/L (40 mg/dL) throughout the hospitalization (Hypo_Severe_). The 3.9 mmol/l threshold is consistent with the typical threshold for treatment in the hospital and both 3.9 mmol/L and 2.2 mmol/L are definitions used by published studies of hospitalized patients and glucometric tools [5, 22–24].

### Glucose management

At the study institution, patients with diabetes are generally managed with subcutaneous basal bolus insulin. Bolus insulin (lispro or aspart) is administered using order panels that specify low, standard, or high dose, corresponding to 1 unit per 20, 10, or 5 g of carbohydrates and 1 unit per 100, 50 or 25 mg/dL above 150 mg/dL respectively. Target glucose range for inpatients is 140–180 mg/dL. Orders for point of care glucose monitoring prior to meals and bedtime accompany all subcutaneous insulin orders, with additional standing orders for glucose monitoring as needed for suspected hypoglycemia or hyperglycemia. Hospital guidelines provide tiered recommendations for management of hypoglycemia according to its severity, which includes oral carbohydrate administration (15–30 g) whenever possible and intravenous dextrose (12.5–25 g of 50% dextrose) when a patient is unable to eat or is not neurologically intact. Follow-up glucose measurement is obtained and re-treatment occurs every 15 min until resolution of hypoglycemia. Point of care glucose was monitored using exact time stamps via the Nova StatStrip^®^ glucometer. Meters are docked and downloaded at least daily and populate into to the electronic medical record.

### Analysis

Continuous variables with normal distribution were reported as mean (standard deviation) and differences between groups were determined using the analysis of variance across groups and unpaired Student’s T-test for comparisons between two groups. Continuous variables with non-normal distribution were reported as median (interquartile range) and analyzed using the Wilcoxon rank-sum test. Dichotomous variables were reported as number (percentage) and differences between groups were assessed using Chi square test. Statistical significance was considered at the threshold p-value less than 0.05.

Multivariable logistic regression was performed for hypoglycemia occurrence as the dependent variable, and BB type (carvedilol, SBB, or none) as the primary independent variable. Covariates were selected based on previous knowledge as well as availability within the electronic medical record [25]. Separate models analyzing hospital mortality as dependent variables and hypoglycemia as the primary independent variable were created for carvedilol, SBB, or non-BB recipients. Analyses were performed using JMP 10.0 software.

## Results

### Patient characteristics

A total of 13,423 unique admissions met the initial inclusion criteria, of which 816 were patients who started BB after admission, 400 patients who switched BB after admission, and 171 patients who received nonselective BB other than carvedilol were excluded (patients may have been excluded for more than one reason). This left a final sample size of 1020 carvedilol, 886 SBB, and 10,216 non-BB recipients. Study population characteristics are shown in Table 1. BB recipients were older, less likely to be male or be on a surgery service, more likely to be on cardiac service, and had higher creatinine, hospital length of stay and frequency of basal insulin use (carvedilol 27%, SBB 33%, no BB 4.9%, p < 0.0001 by ANOVA), sulfonylurea/glinides, other cardiac medications and heart failure. Carvedilol users were less likely to be Caucasian than SBB or non-BB users while SBB were more likely to receive other diabetes medications.

**Table 1:** Baseline characteristics by BB status

	No BB (N = 10,216)	Carvedilol (N = 1020)	SBB (N = 886)	p-value
Age, years	60 (15)	64 (13)	65 (13)	< 0.0001
Male, N (%)	4986 (49)	381 (37)	383 (43)	< 0.0001
Race, N (%)				< 0.0001
Caucasian	7422 (73)	628 (62)	689 (78)	–
African	2293 (22)	357 (35)	174 (20)	–
Asian	121 (1.2)	10 (1.0)	4 (0.45)	–
American Indian	20 (0.20)	1 (0.10)	0 (0)	–
Middle Eastern	28 (0.27)	2 (0.20)	2 (0.23)	–
Other	332 (3.3)	22 (2.2)	17 (1.9)	–
Body mass index, kg/m^2^	33.1 (9.4)	33.7 (9.6)	33.4 (9.6)	0.13
Surgery service, N (%)	2837 (28)	72 (7.1)	140 (16)	< 0.0001
Cardiac service, N (%)	1330 (13)	401 (39)	352 (30)	< 0.0001
Hemoglobin A1c, %	7.98 (2.53) *N *= *1704*	7.71 (2.14) *N *= *186*	7.78 (2.30) *N *= *113*	0.30
Admission creatinine, μmol/L	1.40 (1.50)	2.24 (2.14)	1.63 (1.46)	< 0.0001
Hospital length of stay, days	4 (3–7)	6 (4–10)	6 (4–11)	< 0.0001
Basal insulin at admission, N (%)	500 (4.9)	272 (27)	292 (33)	< 0.0001
Heart failure, N (%)	461 (4.5)	814 (80)	459 (52)	< 0.0001
Sulfonylurea/glinide	25 (0.24)	40 (3.9)	31 (3.5)	< 0.0001
Other glucose lowering agents (non-insulin, non-sulfonylurea/glinide)	67 (0.66)	95 (0.93)	61 (6.9)	< 0.0001
ACE inhibitor/ARB	171 (1.7)	515 (50)	278 (31)	< 0.0001
Statin	340 (3.3)	692 (68)	395 (45)	< 0.0001
Aspirin	361 (3.5)	698 (68)	397 (45)	<0.0001
Mean glucose (mmol/L)				
Admission	175 (95)	175 (92)	168 (87)	0.11
24 h	167 (61)	167 (62)	165 (59)	0.75
72 h	164 (47)	164 (47)	162 (46)	0.51
Hypoglycemia, N (%)				
< 3.9 mmol/L (70 mg/dL), first 24 h	497 (4.9)	138 (13.5)	151 (17)	< 0.0001
< 3.9 mmol/L (70 mg/dL), during hospital stay	1194 (12)	393 (38)	441 (50)	< 0.0001
< 2.2 mmol/L, (40 mg/dL), during hospital stay	132 (1.3)	47 (4.6)	43 (4.9)	< 0.0001
Events < 3.9 mmol/L (70 mg/dL)/day during hospital stay	0 (0.0)	0 (0.17)	0 (0.025)	< 0.0001
Glucose coefficient of variation (%)				
24 h	0.22 (0.13)	0.26 (0.15)	0.26 (0.15)	< 0.0001
72 h	0.24 (0.11)	0.29 (0.13)	0.28 (0.12)	< 0.0001

Continuous variables that are normally distributed are reported as mean (standard deviation) and analyzed with the analysis of variance. Continuous variables that are not normally distributed are reported as median (interquartile range [IQR]) and analyzed using Wilcoxon Rank-sum. Dichotomous variables are presented using number (percentage) and analyzed using the Chi-square test

*BB* beta blocker, *SBB* selective beta blocker

Admission glucose and mean glucose at 24 and 72 h were similar in carvedilol, SBB, and non-BB recipients (Table 1). Glucose coefficient of variation at 24 and 72 h was higher in carvedilol and SBB patients than non-BB recipients. Unadjusted frequency of Hypo_1day_, Hypo_T_, and Hypo_Severe_ were more common in carvedilol and SBB patients than non-BB recipients.

### Relationship between BB and hypoglycemia

The unadjusted odds of all 3 definitions of hypoglycemia were increased for patients receiving carvedilol or SBB compared to no BB use (Table 2). Furthermore, the unadjusted odds of Hypo_1day_ and Hypo_T_, but not Hypo_Severe_ were higher in SSB compared to carvedilol recipients.

**Table 2:** Relationship between beta blocker use and hypoglycemia

	Overall				No basal insulin			Basal insulin		
	OR	95% CI	p-value	p-value interaction^	OR	95% CI	p-value	OR	95% CI	p-value
Odds ratio for glucose < 3.9 mmol/L (70 mg/dL) within 24 h										
Unadjusted model										
Carvedilol vs. none	3.01	2.48–3.71	< 0.0001	–	2.56	1.95–3.37	< 0.0001	0.91	0.65–1.27	0.58
SBB vs. none	4.02	3.30–4.89	< 0.0001		4.96	3.89–6.33	< 0.0001	0.62	0.44–0.88	0.008
SBB vs carvedilol	1.32	1.03–1.70	0.03		1.94	1.39–2.70	0.0001	0.68	0.46–1.02	0.06
Adjusted model										
Carvedilol vs. none	1.45	1.05–2.01	0.0245	< 0.0001	2.15	1.38–3.37	0.0007	1.18	0.74–1.90	0.49
SBB vs. none	1.78	1.31–2.40	0.0002		4.29	2.96–6.20	< 0.0001	0.82	0.51–1.32	0.42
SBB vs. carvedilol	1.22	0.87–1.72	0.25		1.99	1.28–3.09	0.0023	0.70	0.41–1.18	0.18
Odds ratio for glucose < 3.9 mmol/L (70 mg/dL) overall										
Unadjusted model										
Carvedilol vs. none	4.72	4.10–5.42	< 0.0001	–	3.39	2.82–4.09	< 0.0001	1.05	0.72–1.51	0.81
SBB vs. none	7.49	6.48–8.66	< 0.0001		6.30	5.25–7.56	< 0.0001	0.91	0.64–1.29	0.58
SBB vs. carvedilol	1.59	1.32–1.90	< 0.0001		1.86	1.46–2.35	< 0.0001	0.87	0.58–1.30	0.48
Adjusted model										
Carvedilol vs. none	2.56	1.94–3.37	< 0.0001	<0.0001	6.30	4.59–8.65	< 0.0001	1.03	0.65–1.63	0.91
SBB vs. none	2.61	2.05–3.33	< 0.0001		8.69	6.57–11.5	< 0.0001	0.86	0.56–1.31	0.48
SBB vs. carvedilol	1.02	0.77–1.35	0.89		1.38	1.02–1.86	0.03	0.84	0.52–1.36	0.47
Odds ratio for glucose < 2.2 mmol/L (40 mg/dL) overall										
Unadjusted model										
Carvedilol vs. none	3.69	2.62–5.18	< 0.0001	–	2.63	1.50–4.60	0.0007	1.04	0.65–1.64	0.88
SBB vs. none	3.90	2.74–5.54	< 0.0001		6.12	3.91–9.58	< 0.0001	0.45	0.25–0.80	0.007
SBB vs. carvedilol	1.06	0.69–1.61	0.80		2.33	1.23–4.43	0.0098	0.43	0.23–0.81	0.009
Adjusted model										
Carvedilol vs. none	1.68	1.03–2.74	0.04	< 0.0001	3.21	1.49–6.91	0.0029	1.11	0.58–2.10	0.76
SBB vs. none	1.70	1.06–2.75	0.03		6.11	3.37–11.1	< 0.0001	0.56	0.26–1.20	0.13
SBB vs. carvedilol	1.10	0.59–1.75	0.97		1.90	0.90–4.02	0.09	0.50	0.22–1.14	0.10

All adjusted models adjusted for age, gender, race, body mass index*, surgery service, admission glucose*, admission creatinine*, basal insulin use, heart failure, hospital length of stay, cardiovascular service, statin, aspirin, ACE/ARB, ^basal insulin by BB type interaction. * log transformed values

*BB* beta blocker, *SBB* selective beta blocker

For all adjusted models, there was a strong interaction between basal insulin use and BB type and this interaction term was therefore included in all models. All logistic regression models were also adjusted for age, gender, race, body mass index, surgery service, admission glucose, admission creatinine, heart failure, and basal insulin at admission. The adjusted odds of Hypo_1day_ (OR 4.66, 95% confidence interval [CI] 3.61–6.01, p < 0.0001), Hypo_T_ (OR 12.1, 95% CI 9.86–14.95, p < 0.0001), and Hypo_Severe_ (OR 3.56, 95% CI 2.41–5.26, p < 0.0001) was increased in basal insulin nonusers compared to non-uers. The adjusted odds of Hypo_1day_ were greater for carvedilol (odds ratio [OR] 1.45, 95% CI 1.05–2.01, p = 0.0245), and SBB (OR 1.78, 95% CI 1.31–2.40, p = 0.0002) compared to no BB. There was no difference in odds for SBB compared to carvedilol (OR 1.22, 95% CI 0.87–1.72, p = 0.25). The p-value for interaction between BB category and basal insulin use was < 0.0001.

For Hypo_T_, the fully adjusted odds of hypoglycemia were greater for carvedilol (OR 2.56, 95% CI 1.94–3.37, p < 0.0001) and SBB (OR 2.61, 95% CI 2.05–3.33, p < 0.0001) vs. no BB. Further, the odds were similar for SBB vs. carvedilol (OR 1.02, 95% CI 0.77–1.35, p = 0.89). The p-value for interaction between BB category and basal insulin use was < 0.0001.

For Hypo_Severe_, the fully adjusted odds of hypoglycemia were greater for carvedilol (OR 1.68, 95% CI 1.03–2.74, p = 0.04) and SBB (OR 1.70, 95% CI 1.06–2.79, p = 0.03) compared to no BB. The odds were similar for SBB vs. carvedilol (OR 1.10, 95% CI 0.59–1.75, p = 0.97). The p-value for interaction between BB category and basal insulin use was < 0.0001.

Due to the interaction by basal insulin use, separate models were created for basal insulin users and non-users. For Hypo_1day_ the fully adjusted odds of hypoglycemia were greater for basal insulin non-users but not for basal insulin users (Table 2). Moreover, there were greater odds of hypoglycemia associated with SBB vs. carvedilol in basal insulin non-users (OR 1.99, 95% CI 1.28–3.09, p = 0.0003) but not in users (OR 0.70, 95% CI 0.41–1.18, p = 0.46). Likewise, for Hypo_T_ the fully adjusted odds of hypoglycemia were greater for basal insulin non-users but not for basal insulin users. Further, there was a greater odds of hypoglycemia associated with SBB vs. carvedilol in basal insulin non-users (OR 1.38, 95% CI 1.02–1.86, p = 0.03) but not in users (OR 0.84, 95% CI 0.52–1.36, p = 0.47). Similarly, for Hypo_Severe_ the fully adjusted odds of hypoglycemia were greater for basal insulin non-users but not for basal insulin users. The odds of hypoglycemia associated with SBB vs. carvedilol was not significantly different in basal insulin non-users (OR 1.90, 95% CI 0.90–4.02, p = 0.09) or users (OR 0.50, 95% CI 0.22–1.14, p = 0.10).

Due to the observation that patients with heart failure were more likely to receive carvedilol compared to SBB, a sensitivity analysis was conducted among heart failure subgroups (Additional file 1: Table S1). In fully adjusted models, both carvedilol and SBB were associated with increased odds of Hypo_1day_, Hypo_T_, and Hypo_Severe_ in patients without heart failure, but not in patients with heart failure. There was no significant difference in odds of any of the hypoglycemia measures in carvedilol vs. SBB recipients in either subgroup.

### Relationship between hypoglycemia and hospital mortality

Hypo_1day_ was associated with increased adjusted odds of in-hospital mortality in non-BB recipients (OR 2.10, 95% CI 1.15–3.86, p = 0.016) but not among carvedilol (0.79, 95% CI 0.15–4.10, p = 0.78) or SBB (OR 1.67, 95% CI 0.54–5.16, p = 0.37) recipients (Table 3).

**Table 3:** Relationship between hypoglycemia and mortality in beta blocker subgroups

	Non-recipients of beta blockers			Carvedilol recipients			Selective beta blocker recipients		
	OR	95% CI	p-value	OR	95% CI	p-value	OR	95% CI	p-value
Odds ratio for mortality in patients with vs. those without hypoglycemia (< 3.9 mmol/L) within 24 h of admission									
Unadjusted model	2.40	1.56–3.71	< 0.001	0.83	0.25–2.81	0.77	1.60	0.74–3.46	0.23
Adjusted model	2.10	1.15–3.86	0.016	0.79	0.15–4.10	0.78	1.67	0.54–5.16	0.37
Odds ratio for mortality in patients with vs. those without hypoglycemia (< 3.9 mmol/L) overall									
Unadjusted model	2.21	1.60–3.04	< 0.0001	1.18	0.54–2.60	0.68	2.17	1.08–4.37	0.03
Adjusted model	1.80	1.16–2.80	0.009	1.55	0.49–4.95	0.46	4.89	1.76–13.56	0.002
Odds ratio for mortality in patients with vs. those without hypoglycemia (< 2.2 mmol/L) overall									
Unadjusted model	4.63	2.52–8.52	< 0.0001	1.76	0.40–7.67	0.45	6.42	2.73–15.1	< 0.0001
Adjusted model	3.74	1.48–9.46	0.005	1.94	0.36–10.3	0.44	10.6	3.27–34.3	< 0.0001

Adjusted model includes age, gender, race, body mass index*, surgery service, admission glucose*, admission creatinine*, hospital length of stay*, basal insulin, heart failure, cardiovascular service, statin, aspirin, ACE/ARB and hypoglycemia (< 70 mg/dL within 24 h, < 70 during entire hospitalization, or < 40 mg/dL during entire hospitalization. * log transformed values

In contrast, Hypo_T_ was associated with higher adjusted odds of in-hospital mortality in both non-BB (OR 1.80, 95% CI 1.16–2.80, p = 0.009) and SBB (OR 4.89, 95% CI 1.76–13.6, p = 0.002), but not carvedilol (OR 1.55, 95% CI 0.49–4.95, p = 0.46) recipients (Table 3).

In a separate model that included only BB recipients, there was no interaction between hypoglycemia (< 3.8 mmol/L) and BB type on the odds of mortality. However, data are not shown due to unstable estimates.

Likewise, Hypo_Severe_ was associated with increased adjusted odds of in-hospital mortality in both non-BB (OR 3.74, 95% CI 1.48–9.46, p = 0.005), and SBB (OR 10.6, 95% CI 3.27–34.3, p < 0.0001), but not carvedilol (OR 1.94, 95% CI 0.36–10.3, p = 0.44) recipients.

There was no significant interaction by basal insulin use in these models. However, these models demonstrated unstable estimates and therefore were not presented.

## Discussion

### Major findings

In this analysis, the adjusted odds of hypoglycemia within the first 24 h of admission and total or severe hypoglycemia throughout the admission were increased with BB use. However, there was a significant interaction by basal insulin use such that hypoglycemia was only increased in patients who were not using basal insulin. In addition the odds of hypoglycemia were increased with SBB compared to carvedilol, again only in basal insulin users. Carvedilol and SBB were associated with increased risk of hypoglycemia in patients without heart failure but not in patients with heart failure. Finally, hypoglycemia was associated with increased odds of mortality in BB non-recipients and SBB recipients but not with carvedilol use.

### BB and hypoglycemia risk

There are few data describing risk of hypoglycemia among hospitalized BB recipients who are receiving insulin. A recent large inpatient analysis identified risk factors for hypoglycemia, but did not report BB use [26]. Cardona and colleagues [27] did not identify a difference in risk according to BB use, but the study was much smaller (N = 250). In the ambulatory setting, there is also low quality evidence assessing the risk of hypoglycemia with BB use [12]. A recent study did not find increased readmission rate for elderly nursing home patients after acute myocardial infarction with nonselective BB compared to SBB [28]. Among insulin- or sulfonylurea-requiring patients, SBB was not associated with increased risk of serious hypoglycemia compared to nonusers [29, 30]. Similarly, in this study, BB use was associated with hypoglycemia among basal insulin nonusers, but not among basal insulin users. While we can not determine the extent or duration of insulin use prior to admission, it is possible that basal insulin use represents patients with previous or longer duration of insulin use. It has been reported that duration of insulin use [31], and increasing complexity of insulin regimens [32], are associated with increased risk of hypoglycemia and hypoglycemia unawareness. In rat models of cardiac ischemia and reperfusion, hearts from animals with diabetes but not hearts from animals without diabetes were amenable to cardioprotection due to ischemic preconditioning during hypoglycemia [33]. Thus BB use may not be relevant in patients with long-standing insulin use, some of whom may already have loss of early counterregulatory responses or frank hypoglycemia unawareness [34]. Moreover, while acute BB administration may attenuate the loss of counterregulatory responses induced by antecedent hypoglycemia, the long-term effects are less clear, and in this study, only chronic BB use was included [13]. Thus, more research is needed to investigate the effects of long-term BB use and its interaction with insulin use.

### Role of BB type

The odds of hypoglycemia were slightly greater with SBB than with carvedilol in this study. This finding remains unexplained, but could be related to differences in β-receptor and α-receptor activity and the attendant autonomic responses to hypoglycemia [35]. This was the rationale for excluding other BB, which were few in number and had other unique characteristics. For example, propranolol, also a nonselective BB, has been reported to result in adverse hemodynamic effects during hypoglycemia, possibly due to unopposed α-1 receptor activity [10, 11], and more severe hypoglycemic events compared to SBB [30, 36]. Therefore, SBB are generally recommended in patients requiring concomitant insulin. However, the current results suggest that this recommendation may not apply to all nonselective BBs. Carvedilol has α-1 receptor blocking effects and may therefore mitigate the adverse effects observed with propranolol. While previous studies demonstrate that adrenergic responses to experimentally induced hypoglycemia are mediated by the β-receptors (not α-receptors), responses to experimentally derived hypoglycemia may not adequately reflect counterregulatory responses in chronic use or in severe illness [37]. For example, multiple stressors can cause opioid receptor activation, in part via α-1 receptors in the adrenal gland, which in turn may impair hypoglycemia counterregulation [38, 39]. Moreover, carvedilol use is associated with lower hypoglycemia symptom scores and a favorable metabolic profile compared to metoprolol [19], which may influence insulin requirements and hypoglycemia [40, 41]. In this study, carvedilol and SBB recipients were more likely to receive basal insulin than non-BB users. However, it is unlikely that basal insulin use influenced BB prescribing practices, as insulin use was similar among carvedilol or SBB recipients. Finally, it is possible that residual confounding by indication may explain the results, particularly since tolerability of carvedilol may lessen with more severe illness, such as advanced heart failure. A separate analysis by heart failure status did not confirm the increase in risk of hypoglycemia in patients without heart failure receiving SBB compared to carvedilol but the sample size may be too small to draw conclusions (N = 206 carvedilol, N = 427 SBB).

### BB and heart failure

Carvedilol and SBB decrease long-term mortality in heart failure [42, 43]. Despite this, clinicians have historically been hesitant to prescribe BB to patients with diabetes and heart disease due to concern for adverse effects [44]. In the present study, carvedilol and SBB were associated with increased risk of hypoglycemia in patients without heart failure but not in patients with heart failure. The reduction in hypoglycemia-associated autonomic failure due to BB use offers a potential mechanism [14]. A secondary analysis of the ACCORD trial found increased mortality in patients with cardiovascular disease and diabetes treated with BB, however it did not identify the types of BB used by study participants [8]. Further research is needed to determine the relationship of beta blockade and hypoglycemia in patients with comorbid diabetes and HF.

### Mortality

Hypoglycemia was associated with increased mortality in non-BB patients and SBB users, but not in carvedilol users. This finding is in line with the findings observed for hypoglycemia, though is insufficient to establish a causal relationship between hypoglycemia and mortality. In the ambulatory environment, BB use is associated with mortality and cardiovascular events in patients with diabetes [8, 18], though not in older studies [45], possibly due to better control of other risk factors in more recent literature. Nevertheless, BBs appeared to be protective of the increase in all-cause and cardiovascular death observed with intensive glycemic control in the ACCORD trial [15]; however BB type and insulin use was not analyzed separately in any of these studies. The present findings are consistent with a recent observational study of outpatients, which found that SBB but not nonselective BB were associated with a possible reduction in mortality in patients with episodes of hypoglycemia [46]. In another study of older nursing home patients discharged after myocardial infarction, there was no difference in mortality between patients prescribed carvedilol and those prescribed metoprolol [28]. Insulin induced hypoglycemia has been postulated to elicit an adverse sympathoadrenal response that predisposes to arrhythmia and death. This response is blunted by recurrent antecedent hypoglycemia or BB administration [47]. Thus, the sympathoadrenal, and presumably the cardiovascular response to hypoglycemia may rely upon a complex interaction of multiple factors, including duration of diabetes, insulin exposure and BB use and type, and requires further study. In contrast to hypoglycemia incidence, basal insulin use was not an effect modifier for BB associated mortality though effect estimates were unstable and definitive conclusions could not be drawn.

The present study has several limitations, owing to its retrospective nature and as a result can not be used to determine causality. The duration of diabetes, comorbidities other than renal function and etiology of death was unknown. A diagnosis of diabetes was not an inclusion criterion due to the limitations of using diagnosis codes for identifying at risk patients and because patients undergoing glucose monitoring with insulin orders were considered a more relevant population. The exclusion of patients who switched BB limits the external applicability of these results, particularly since the reason for switching was unknown. However, including such patients would not allow for the comparison of effects of specific agents. Other nonselective BB were excluded because of very small numbers and/or unique mechanisms of action. It is important to note that β-1 selectivity theoretically decreases with increasing doses but doses were not collected for this study. The timing of hypoglycemia with respect to insulin doses and actual doses were also unavailable, though all patients had orders for insulin therapy. In addition, this study was unable to assess symptoms of hypoglycemia. In a previous study of 250 insulin requiring hospitalized patients, 45% of patients who had a glucose < 3.8 mmol/L were asymptomatic [27]. Despite these limitations, this is the largest study of BB use and hypoglycemia risk in hospitalized patients to date.

## Conclusions

This study indicates that BB use is associated with increased odds of hypoglycemia in hospitalized insulin requiring patients but this was limited to patients who were not receiving basal insulin. Among basal insulin nonrecipients, hypoglycemia risk was greater with SBB compared to carvedilol. Carvedilol and SBB were associated with increased risk of hypoglycemia in patients without heart failure but not in patients with heart failure. Moreover, hypoglycemia is associated with increased hospital mortality among patients receiving SBB, those receiving no BB, but not among patients receiving carvedilol. Further study is needed to understand the role of previous insulin exposure and hypoglycemia associated with BB use and BB type.

## Supplementary information

**Additional file 1: Table S1.** Relationship between BB and hypoglycemia in heart failure subgroups.

## Data Availability

In accordance with institution policy on sharing data and research resources, the final research data from this study may be made available for research purposes under a limited data use agreement specifying criteria for data access, conditions for research use, privacy and confidentiality standards to ensure data security and prohibitions for manipulating data for the purposes of identifying subjects.

## Abbreviations

BB: beta blocker
SBB: selective beta blocker
ICD: International Classification of Diseases
Hypo_1day_: any glucose < 3.9 mmol/L within 24 h of admission
Hypo_T_: any glucose < 3.9 mmol/L throughout hospitalization
Hypo_severe_: any glucose < 2.2 mmol/L throughout hospitalization
OR: odds ratio
CI: confidence interval
