# Supplementary material for: Effect of beta blocker use and type on hypoglycemia risk among hospitalized insulin requiring patients
Source: Cardiovasc Diabetol. 2019 Nov 27;18:163. doi: 10.1186/s12933-019-0967-1 (PMC6882013; doi:10.1186/s12933-019-0967-1)
Supplement: Supplementary file 1 — Additional file 1: Table S1. Relationship between BB and hypoglycemia in heart failure subgroups. [file 12933_2019_967_MOESM1_ESM.docx]

**Appendix**

**Table S1.** Relationship between BB and Hypoglycemia in Heart Failure Subgroups

|  | **No Heart Failure** | | | **Heart Failure** | | |
| --- | --- | --- | --- | --- | --- | --- |
|  | **OR** | **95% CI** | **p-value** | **OR** | **95% CI** | **p-value** |
| **Odds Ratio for Glucose <3.9 mmol/L (70 mg/dl) within 24 hours** | | | | | | |
| Unadjusted model  Carvedilol vs. none  SBB vs. none  SBB vs. Carvedilol | 8.48  7.42  0.88 | 6.18-11.6  5.86-9.40  0.60-1.27 | <0.0001  <0.0001  0.48 | 0.77  0.69  0.90 | 0.53-1.10  0.45-1.06  0.60-1.34 | 0.15  0.09  0.61 |
| Adjusted Model  Carvedilol vs. none  SBB vs. none  SBB vs. Carvedilol | 2.34  3.80  1.20 | 1.51-3.63  1.97-3.97  0.72-1.97 | <0.0001  <0.0001  0.49 | 0.81  0.71  0.88 | 0.49-1.36  0.39-1.30  0.51-1.51 | 0.43  0.27  0.64 |
| **Odds Ratio for Glucose <3.9 mmol/L (70 mg/dl) overall** | | | | | | |
| Unadjusted Model  Carvedilol vs. none  SBB vs. none  SBB vs. Carvedilol | 24.7  26.9  1.09 | 17.9-34.1  21.3-34.0  0.74-1.61 | <0.0001  <0.0001  0.66 | 1.09  0.87  0.80 | 0.85-1.41  0.65-1.17  0.61-1.03 | 0.50  0.35  0.08 |
| Adjusted Model  Carvedilol vs. none  SBB vs. none  SBB vs. Carvedilol | 3.82  5.06  1.32 | 2.60-5.61  3.75-6.81  0.85-2.06 | <0.0001  <0.0001  0.21 | 1.40  1.01  0.73 | 0.89-2.19  0.61-1.69  0.46-1.14 | 0.15  0.96  0.17 |
| **Odds Ratio for Glucose <2.2 mmol/L (40 mg/dl) overall** | | | | | | |
| Unadjusted Model  Carvedilol vs. none  SBB vs. none  SBB vs. Carvedilol | 10.8  5.08  0.47 | 6.78-17.1  3.26-7.91  0.26-0.85 | <0.0001  <0.0001  0.01 | 0.93  1.30  1.40 | 0.47-1.82  0.64-2.65  0.75-2.63 | 0.83  0.47  0.29 |
| Adjusted Model  Carvedilol vs. none  SBB vs. none  SBB vs. Carvedilol | 2.77  1.57  0.57 | 1.55-4.97  0.85-2.91  0.27-1.21 | 0.0006  0.15  0.14 | 0.85  1.65  1.94 | 0.34-2.13  0.66-4.13  0.85-4.46 | 0.73  0.28  0.12 |

All adjusted models includes age, gender, race, body mass index*, surgery service, admission glucose*, admission creatinine*, basal insulin, cardiovascular service, statin, aspirin, ACE/ARB. *log transformed values.
